# Impact of Visceral Fat on Skeletal Muscle Mass and Vice Versa in a Prospective Cohort Study: The Korean Sarcopenic Obesity Study (KSOS)

**DOI:** 10.1371/journal.pone.0115407

**Published:** 2014-12-17

**Authors:** Tae Nyun Kim, Man Sik Park, Ja Young Ryu, Hae Yoon Choi, Ho Cheol Hong, Hye Jin Yoo, Hyun Joo Kang, Wook Song, Seok Won Park, Sei Hyun Baik, Anne B. Newman, Kyung Mook Choi

**Affiliations:** 1 Division of Endocrinology and Metabolism, Department of Internal Medicine, College of Medicine, Korea University, Seoul, Korea; 2 Department of Internal Medicine, Cardiovascular and Metabolic Disease Center, College of Medicine, Inje University, Busan, Korea; 3 Department of Statistics, College of Natural Sciences, Sungshin Women's University, Seoul, Korea; 4 Sports Medicine, Division of Physical Education, Soonchunhyang University, A-San, Korea; 5 Health and Exercise Science Laboratory, Institute of Sports Science, Department of Physical Education, Seoul National University, Seoul, Korea; 6 Department of Internal Medicine, Pochon CHA University, Pochon, Korea; 7 Department of Epidemiology, University of Pittsburgh, Pittsburgh, Pennsylvania, United States of America; University of Wisconsin, United States of America

## Abstract

**Objectives:**

Sarcopenia and visceral obesity have been suggested to aggravate each other, resulting in a vicious cycle. However, evidence based on prospective study is very limited. Our purpose was to investigate whether visceral fat promotes a decrease in skeletal muscle mass and vice versa.

**Methods:**

We observed changes in anthropometric and body composition data during a follow-up period of 27.6±2.8 months in 379 Korean men and women (mean age 51.9±14.6 years) from the Korean Sarcopenic Obesity Study (KSOS). Appendicular lean soft tissue (ALST) mass was calculated using dual-energy X-ray absorptiometry, and visceral fat area (VFA) was measured using computed tomography at baseline and follow-up examination.

**Results:**

ALST mass significantly decreased, whereas trunk and total fat mass increased in both men and women despite no significant change in weight and body mass index. In particular, women with visceral obesity at baseline had a greater decrease in ALST mass than those without visceral obesity (*P* = 0.001). In multiple linear regression analysis, baseline VFA was an independent negative predictor of the changes in ALST after adjusting for confounding factors including age, gender, life style and body composition parameters, insulin resistance, high sensitivity C-reactive protein and vitamin D levels (*P* = 0.001), whereas the association between baseline ALST mass and changes in VFA was not statistically significant (*P* = 0.555).

**Conclusions:**

This longitudinal study showed that visceral obesity was associated with future loss of skeletal muscle mass in Korean adults. These results may provide novel insight into sarcopenic obesity in an aging society.

## Introduction

Aging is often accompanied by changes in body composition that lead to a shift toward decreased muscle mass and increased fat mass, even in relatively weight-stable, healthy individuals [1], [2]. Both obesity and sarcopenia, the age-related loss of skeletal muscle mass and function, are important causes of frailty, disability, morbidity, and mortality [3]. Diminished skeletal muscle and expanded visceral fat may act synergistically, which may maximize their effects on physical impairments and metabolic disorders [4].

Fat and muscle mass are known to be strongly interconnected from a pathogenic point of view [4]. Previous studies suggested that sarcopenia may aggravate obesity and vice versa. Loss of skeletal muscle induces a 2%–3% decline in basal metabolic rate per decade after the age of 20 years, and a 4% decline per decade after the age of 50 years [5]. In addition, sarcopenia reduces the intensity and duration of physical activity, which results in decreased energy expenditure. These changes may increase the risk of obesity and obesity-related metabolic disorders, such as metabolic syndrome [3]. On the other hand, increased adiposity induces chronic subclinical inflammation, which may contribute to the development and progression of sarcopenia. Dysregulation of adipokines originating from visceral adipose tissue, such as tumor necrosis factor-α (TNF-α), interleukin-6 (IL-6), leptin, and adiponectin, has been reported to influence insulin resistance and growth hormone (GH) secretion, which are closely associated with sarcopenia [6]. Insulin is a pivotal anabolic signal and insulin resistance is regarded to be the main factor that connects obesity and sarcopenia [7].

Although these reports support the hypothesis that sarcopenia and obesity may worsen each other, creating a vicious cycle, previous longitudinal human studies that explore the contribution of visceral fat accumulation to changes in skeletal muscle mass or vice versa are very limited. Furthermore, to the best of author's knowledge, there was no previous study to compare which one among sarcopenia and visceral fat might be an initiating factor after adjusting potential confounding factors.

Our aims in this prospective human study were therefore to examine changes in parameters of obesity and sarcopenia using accurate standard imaging methods, such as computed tomography (CT) and dual-energy X-ray absorptiometry (DXA), and to evaluate if baseline visceral fat area (VFA) are negatively associated with changes in skeletal muscle mass and vice versa.

## Research Design and Methods

### Subjects and data collection

The Korean Sarcopenic Obesity Study (KSOS) is a longitudinal study funded by the Korea Science and Engineering Foundation. This prospective observational cohort study was designed to examine the prevalence of sarcopenia and sarcopenic obesity in Korean adults with (diabetic KSOS cohort) or without diabetes (non-diabetic KSOS cohort) and to evaluate effects of sarcopenia and sarcopenic obesity on metabolic disorders and health outcomes [8], [9]. In this study, we analyzed baseline and follow-up data from the KSOS. Eligible participants (20–86 years) were those in the non-diabetic KSOS cohort; these subjects had no history of any type of diabetes, cardiovascular disease (CVD) (myocardial infarction, unstable angina, stroke or cardiovascular revascularization), stage 2 hypertension (resting blood pressure, ≥160/100 mmHg), malignant disease, or severe renal or hepatic disease. Participants underwent body compositional analysis using precise techniques to measure muscle mass and visceral fat, namely DXA and CT, simultaneously. Medical histories and lifestyle information were collected by personal interview using a detailed questionnaire [10]. Physical activity was classified into two categories: none or regularly. Regular exercise was defined as exercising 30 minutes or more at least three times a week. Complete body composition data were available for 379 participants at baseline and after a mean follow-up of 27.6±2.8 months. All participants provided written informed consent, and the Korea University Institutional Review Board, in accordance with the Declaration of Helsinki of the World Medical Association approved this study protocol.

### Anthropometric and body composition measurements

Height, body weight, and waist circumference were measured following standardized procedures. Body mass index (BMI) was calculated as weight in kilograms divided by the square of height in meters. A whole body DXA scan was performed for each patient to measure both the total and regional components of body composition using fan-beam technology (Hologic Discovery A, Hologic; Bedford, MA, USA). Measurements of both arms, both legs, and the head were separated from trunk measurements using computer-generated default lines with manual adjustment in the anterior view planogram [11]. An experienced medical radiology technician handled the adjustment of all subjects using specific anatomical landmarks (chin, center of the glenohumeral joint, and femoral neck axis). Total, trunk, and appendicular body composition were individually evaluated. Appendicular lean soft tissue (ALST) mass (kg) was defined as the sum of the lean soft tissue masses for the arms and legs, following the method of Heymsfield et al. [12]. Appendicular fat mass (AFM) was calculated as the sum of fat mass from both arms and legs. Abdominal adipose tissue area was quantified by CT (Brilliance 64, Philips Medical Systems, Cleveland, Ohio). Fat area was determined by measuring the mean value of the pixels within the range of −190 to −30 Hounsfield units. Total abdominal fat area (TFA), VFA and subcutaneous fat area (SFA) were measured using a 10-cm CT slice scan image between the fourth and fifth lumbar vertebrae that was obtained during suspended respiration. VFA was calculated by delineating the intra-abdominal cavity at the internal aspect of the abdominal and oblique muscle walls surrounding the cavity and the posterior aspect of the vertebral body. SFA was calculated by subtracting VFA from TFA. Changes in each an anthropometric and body composition measurement between baseline and follow-up were calculated as follow-up value minus baseline value.

### Definitions of sarcopenia and visceral obesity

The predictive equation of Kim et al. [13] was applied to compute the total skeletal muscle mass from ALST mass, using the following equation: [14]

Total skeletal muscle mass (kg) = (1.13×ALST mass)−(0.02×age) + (0.61×sex)+0.97 where sex = 0 for female and sex = 1 for men.

The skeletal muscle index (SMI (%); total skeletal muscle mass (kg)/weight (kg) ×100) was obtained by calculating the total skeletal muscle mass adjusted by weight as described by Janssen et al. [15]. Sarcopenia was defined as an SMI of <2 SD below the sex-specific mean value for young reference group from the entire study population [15]. The cutoff point for sarcopenia was 37.0% in men and 30.5% in women. Visceral obesity was defined as a visceral fat area exceeding 100 cm^2^ in both men and women [16].

### Laboratory measurements

All blood samples were obtained in the morning after a 12-hour overnight fast and were immediately stored at −80°C for subsequent assays. Serum triglycerides and high-density lipoprotein (HDL)-cholesterol levels were determined enzymatically using a chemistry analyzer (Hitachi 747; Tokyo, Japan). A glucose oxidase method was used to measure fasting plasma glucose (FPG), and high-sensitivity C-reactive protein (hsCRP) levels were measured by Latex-enhanced Turbidometric Immunoassay (HiSens hsCRP LTIA; HBI Co., Ltd.) with an interassay coefficient of variation of 7.2%. Insulin resistance was calculated using Homeostasis Model Assessment (HOMA) [17]. Serum 25[OH]D levels were measured using radioimmunoassay kits (DIAsource Diagnostics, Nivelles, Belgium) with quality control materials provided by the manufacturer.

### Statistical analysis

Numerical data are expressed as means ± standard deviations or medians [inter-quartile ranges]. Categorical variables are presented as percentages. Baseline anthropometric, body composition, and metabolic characteristics of participants are presented separately for men and women. Differences in quantitative variables between groups were investigated using the independent two-sample *t*-test or Wilcoxon's rank-sum test, and Pearson's chi-squared test was used to test for differences in the distribution of categorical variables. Paired-sample *t*-test or Wilcoxon's signed-rank test were performed to compare baseline and follow-up levels of anthropometric and body composition parameters. Spearman correlation analysis was performed to determine the relationships between VFA and changes in ALST mass or vice versa. In addition, spearman's partial correlation analysis adjusting for age and gender was performed. Multiple linear regression analyses using changes in VFA or ALST mass as the dependent variable were conducted to determine the relative contributions made by each variable to the outcome variables. Age, sex, smoking, alcohol, physical activity, systolic blood pressure (SBP), diastolic blood pressure (DBP), total cholesterol, triglycerides, HDL-cholesterol, fasting plasma glucose, hsCRP levels, 25[OH]D, BMI, AFM, ALST mass, trunk fat mass, SFA, and VFA at baseline as well as changes in ALST mass, VFA, and body weight were used as independent variables. Statistically significant independent variables were chosen by means of the backward elimination method. All statistical outcomes based on a two-sided test were obtained using SAS for Windows (Version 9.2, SAS Institute Inc., Cary, NC, USA). We regarded a *P*-value <0.05 as statistically meaningful.

## Results

Baseline clinical and metabolic characteristics of the study subjects are shown in Table 1. No difference of total cholesterol, HOMA-IR, and hsCRP was found between men and women. Age, SBP, DBP, triglyceride, and 25[OH]D were higher in men than in women (*P*<0.001, respectively), whereas HDL cholesterol was significantly lower in men than in women (Table 1). When men were compared with women according to baseline anthropometric and body composition indicators, BMI (P = 0.019), weight (*P*<0.001), VFA (*P*<0.001), ALST mass (*P*<0.001), and total skeletal muscle mass (*P*<0.001) were all greater in men than women. SFA and total fat mass were significantly greater in women (*P*<0.001, respectively).

**Table 1:** Baseline clinical and metabolic characteristics of study subjects.

	Men (n = 141)	Women (n = 238)	*P*-value
Age (years)	53.8±13.9	50.8±14.9	0.054
Systolic blood pressure (mmHg)	126.8±12.6	120.5±13.6	<0.001
Diastolic blood pressure (mmHg)	83.5±9.7	77.9±10.2	<0.001
Total cholesterol (mmol/L)	4.8±0.9	4.8±0.9	0.637
HDL cholesterol (mmol/L)	1.3±0.3	1.5±0.3	<0.001
Triglycerides (mmol/L)	1.5 [1.0, 2.1]	1.1 [0.8, 1.6]	<0.001
Fasting plasma glucose (mmol/L)	5.1 [4.8, 5.5]	5.3 [5.0, 5.8]	<0.001
HOMA-IR	2.0 [1.4, 2.8]	1.9 [1.3, 2.7]	0.478
hsCRP (mg/L)	0.5 [0.2, 0.9]	0.4 [0.1, 0.9]	0.135
25[OH]D (nmol/L)	89.3 [65.4, 116.7]	63.3 [47.2, 91.3]	<0.001
Prevalence of visceral obesity (%)	81.6	47.1	<0.001
Prevalence of sarcopenia (%)	4.6	5.6	0.694

HDL, high density lipoprotein; HOMA-IR, homeostasis model assessment of insulin resistance; hsCRP, high-sensitivity C-reactive protein; 25[OH]D, 25-hydroxyvitamin D.

Data are presented as mean ± SD, median [inter-quartile range].

*P*-values were obtained from the independent two-sample *t*-test or Wilcoxon's rank-sum test.

Anthropometric and body composition characteristics of the study participants before and after the follow-up period are presented in Table 2. During that timeframe, most men and women gained fat mass, whereas all body muscle indices decreased significantly. Among the anthropometric and body composition parameters, ALST mass, total skeletal muscle mass, and AFM decreased significantly. On the contrary, trunk and total fat mass increased. However, there were no changes in weight and BMI in either men or women. In addition, VFA did not decrease in men, whereas it tended to be higher than baseline at the study follow-up in women.

**Table 2:** Body composition characteristics of study subjects at baseline and after 2 years.

Characteristics	Men (n = 141)			Women (n = 238)		
	Baseline	Follow-up	*P*-value	Baseline	Follow-up	*P*-value
Body mass index (kg/m^2^)	24.9±2.7	24.7±2.7	0.182	24.1±3.6	24.1±3.8	0.661
Weight (kg)	72.1±10.6	71.6±11.2	0.123	59.2±9.5	59.3±9.8	0.619
Visceral fat area (cm^2^)	138.2 [107.7, 174.6]	138.3 [106.3, 178.1]	0.823	97.3 [60.9, 141.8]	99.9 [67.3, 141.3]	0.082
Appendicular fat mass (kg)	6.4 [5.5, 7.9]	6.0 [5.2, 7.3]	<0.001	10.0 [8.3, 12.2]	9.4 [7.7, 11.1]	<0.001
Trunk fat mass (kg)	6.4 [5.1, 8.2]	6.9 [5.1, 9.1]	<0.001	6.9 [5.2, 9.0]	8.0 [6.0, 10.6]	<0.001
Total fat mass (kg)	14.2 [12.0, 17.2]	14.3 [11.6, 17.6]	0.028	18.2 [15.0, 22.3]	18.3 [15.3, 22.6]	0.001
Appendicular lean soft tissue mass (kg)	26.2 [23.9, 29.1]	25.2 [22.9, 27.4]	<0.001	18.1 [16.8, 20.2]	17.4 [15.9, 18.9]	<0.001
Total skeletal muscle mass (kg)	30.2 [27.6, 33.5]	28.9 [26.3, 31.5]	<0.001	20.5 [19.0, 22.6]	19.6 [17.8, 21.3]	<0.001

Data are presented as mean ± SD, median [inter-quartile range].

*P*-values were obtained from paired-sample *t*-test or Wilcoxon's signed-rank test.

**Figure 1:**
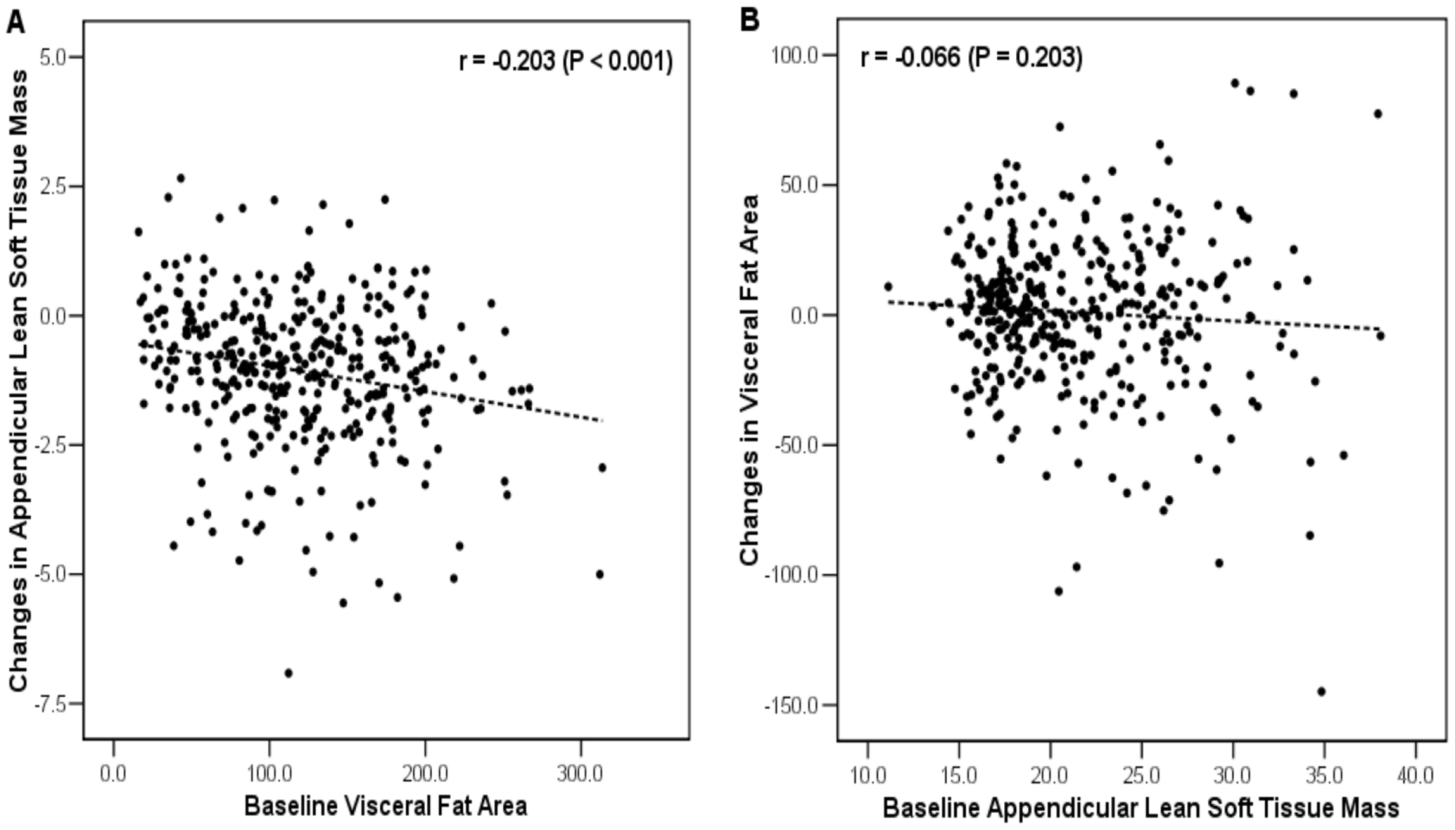
Relationship between baseline visceral fat area and changes in appendicular lean soft tissue mass and vice versa. Scatter plot of baseline visceral fat area (cm^2^) against change in appendicular soft tissue mass (kg) (A) and vice versa (B).

Results of correlation analysis between baseline VFA and changes in ALST mass, and between baseline ALST mass and changes in VFA are presented in Fig. 1. VFA at baseline was negatively associated with changes in ALST mass during follow-up period (*r* = −0.20, *P*<0.001). Adjustment for age and sex did not affect the strength of the association between baseline VFA and changes in ALST mass (*r* = −0.17, *P* = 0.001). However, on the contrary, there were no relationships between baseline ALST mass and changes in VFA (*r* = −0.08, *P* = 0.135).

**Figure 2:**
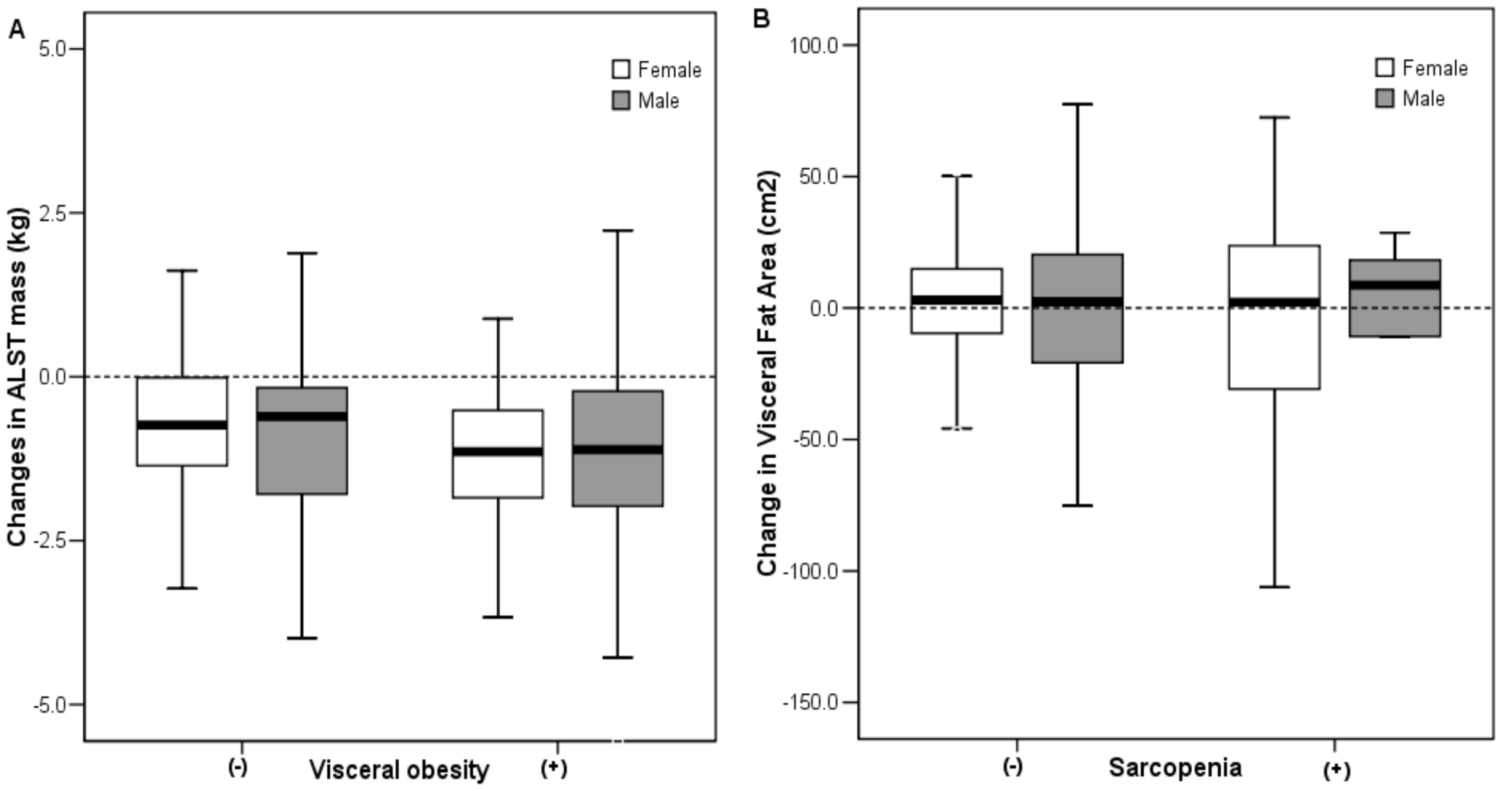
Changes in appendicular lean soft tissue mass in participants classified on the basis of visceral obesity (A) and changes in visceral fat area according to the presence of sarcopenia (B) in men and women. The box plot display the 25^th^, median and 75^th^ percentiles and the minimum and maximum levels as horizontal lines outside the box.

Fig. 2 illustrates the changes in ALST mass or VFA in men and women classified on the presence of visceral obesity or sarcopenia. Women with visceral obesity had more decreased ALST mass than those without visceral obesity (−1.1 [−1.8, −0.5] vs. −0.7 [−1.4, −0.1], *P* = 0.001), whereas, in men, the difference was not statistically significant (−1.1 [−2.0, −0.2] vs. −0.6 [−1.8, −0.1], *P* = 0.361). The difference of the changes in VFA based on the presence of sarcopenia was not significant in both men and women.

Multiple regression analyses were performed using changes in VFA or ALST mass as the dependent variable and clinical, metabolic, and body composition parameters as independent variables (Table 3). Baseline VFA was independently associated with changes in ALST mass (*P* = 0.001) after adjustment for confounding parameters including age, gender, life style and body composition parameters, insulin resistance, high sensitivity C-reactive protein and vitamin D levels. On the other hand, the association between baseline ALST mass and changes in VFA was not statistically significant (*P* = 0.555).

**Table 3:** Independent associations of changes in the appendicular lean soft tissue (ALST) mass and visceral fat area (VFA) and baseline clinical, metabolic and body composition parameters during the 2-year follow-up period according to multiple linear regression analyses.

Dependent variable	Independent variables	Standardized β	*P*-value
Δ ALST mass^a^	Baseline VFA	−0.240	0.001
	Baseline BMI	−0.307	0.001
	Alcohol drinking	−0.091	0.066
	ΔWeight	0.373	<0.001
	Baseline Subcutaneous fat area	−0.401	<0.001
	Baseline trunk fat mass	0.655	<0.001
ΔVFA^b^	Smoking	−0.131	0.040
	Total cholesterol	−0.104	0.035
	ΔWeight	0.517	<0.001
	Baseline BMI	0.220	0.024
	Baseline appendicular fat mass	−0.312	<0.001
	Baseline trunk fat mass	−0.177	0.048
	Baseline ASM	−0.036	0.555

Variables included in the model were age, sex, smoking, alcohol, physical activity, SBP, DBP, total cholesterol, triglycerides, HDL-cholesterol, fasting plasma glucose, HOMA-IR, hsCRP levels, 25[OH]D, BMI, ALST mass, appendicular fat mass, trunk fat mass, subcutaneous fat area, and VFA at baseline as well as change in ALST mass, VFA and weight.

a Baseline and changes in ALST mass among independent variables ware excluded in the model.

b Baseline and changes in VFA among independent variables ware excluded in the model.

Statistically significant variables were selected via the backward elimination method.

## Discussion

Our study substantially supports the hypothesis of the contribution of increased visceral fat to future loss of muscle mass. Additionally, for the first time, we evaluated if baseline visceral fat area is negatively associated with changes in skeletal muscle mass and vice versa. We found that baseline visceral fat area measured using CT influenced changes in ALST mass, although baseline ALST mass did not predict changes in VFA during the follow-up period. This provides an evidence that visceral obesity might be an influencing factor on skeletal muscle mass. Furthermore, these results suggest that prevention of visceral obesity may decrease the loss of skeletal muscle mass.

In this longitudinal study of apparently healthy Asian men and women with a wide range of age, we found that total and trunk fat mass increased significantly in both men and women, whereas appendicular muscle and appendicular fat mass decreased without parallel changes in BMI and weight. These findings showed that changes in body composition during aging were loss of muscle mass and centralization of body fat. Recently, cross-sectional study showed that a new anthropometric measure based on waist circumference (A Body Shape Index, ABSI) was negatively associated with fat-free mass index, both in men and women [18]. In addition, the lower-ABSI women and men showed a significant greater fat-free mass index than the higher-ABSI groups [18]. Our longitudinal study clearly demonstrated an independent and negative association between baseline visceral fat and changes in skeletal muscle mass.

The rapid increase in numbers of older people in industrialized countries imposes serious burden on health care system. Aging is associated with critical changes in body composition and systemic metabolism. Peak skeletal muscle mass is generally attained with the first three decades of life. Thereafter, there is a progressive reduction in muscle mass of about 40% accompanied by a rise in fat mass between the ages of 20 and 70 years [3]. Sarcopenia and obesity may act synergistically to erode functional capacity and health status in elderly individuals to a greater extent than either disorder alone [19]. Baumgartner et al. showed that individuals with sarcopenic obesity at baseline were two to three times more likely to report onset of disability during follow-up than lean sarcopenic or non-sarcopenic obese subjects [20]. We observed in a previous study that visceral fat to thigh muscle area (VMR), a single indicator of sarcopenic obesity, was independently associated with metabolic syndrome [21]. Expanded fat mass may exacerbate sarcopenia because chronic lipid accumulation has a deleterious effect on the incorporation of amino acids and muscle protein synthesis [22]. Furthermore, chronic high fat feeding has been shown to impair the ability of skeletal muscle to hypertrophy in response to increased mechanical load in mice [23]. Pasco et al. reported that over a period of 5 years, an increase in body fat mass has been accompanied by a decline in both lean mass and bone mass [24]. Koster et al. recently reported that greater total body fat mass measured using DXA at baseline was associated with a greater decline in leg lean mass in both men and women [25]. However, visceral obesity, which is known to be most closely associated with metabolic deterioration and catabolic cytokines, was not measured. Furthermore, they did not examine whether baseline muscle mass can affect visceral fat. Interestingly, the present study first showed that baseline VFA measured using CT influenced changes in ALST mass, although baseline ALST mass did not predict changes in VFA during the follow-up period.

The etiology of sarcopenia is multi-factorial, and includes insulin resistance, inflammation, oxidative stress, and hormonal changes [26]. Recent studies have established that obesity is a state of low-grade, chronic inflammation that is closely associated with insulin resistance [11]. Increased intramyocellular fat content in obesity and aging interferes with the phosphorylation pathway of the insulin receptor and GLUT-4 translocation [27]. Recent evidence suggests that insulin resistance is associated with the development of sarcopenia through alteration of mitochondrial function [28]. Furthermore, enlarged adipocytes and activated macrophages in visceral adipose tissue produce pro-inflammatory adipokines [29]. Schaap et al. reported that higher levels of inflammatory markers, especially TNF-α and its soluble receptors, were associated with a greater 5-year decline in thigh muscle area in the Health, Aging, and Body Composition study [30]. IL-6 and CRP, known as “geriatric cytokines”, increases during the aging process, as does visceral obesity [3]. Haddad et al. reported that IL-6 infusion resulted in atrophy of the tibialis anterior muscle in mice [31]. In the Longitudinal Aging Study Amsterdam, higher levels of IL-6 and CRP were associated with a greater decline in muscle strength over 3 years [32]. A 12-week combined resistance and aerobic exercise program increased strength by an average of 38% along with a significant reduction in CRP levels [33]. In our previous study analyzing cross-sectional KSOS data, we found that hsCRP levels were negatively correlated with SMI and positively correlated with VFA in both men and women [34]. On the other hand, several studies have shown that vitamin D deficiency is associated with various measures of obesity, including waist circumference [35], and increases the risk of sarcopenia [36]. We recently observed that serum 25-hydroxyvitamin D (25[OH]D) levels were independently associated with sarcopenic obesity in Korean men [34]. Interestingly, the present study revealed that baseline VFA were independently associated with changes in ALST mass even after adjusting for the possible mediating factors, such as hsCRP, HOMA-IR and 25[OH]D levels during the follow-up period.

Intimate tissue cross-talk between adipose tissue and skeletal muscle may be essential for the integrated control of systemic physiology in humans. Adipose tissue-derived adipokines have been established as important regulators of insulin resistance and metabolic homeostasis [29]. Carbo et al. reported that administration of leptin, a prototype of adipokine, might decrease the rate of myofibrillar protein synthesis in skeletal muscle without changes in circulating insulin levels [37]. Attenuation of adiponectin contributes to the development of insulin resistance and perpetuates chronic inflammation in muscle tissue [26]. Like adipokines, recent studies found that skeletal muscle also secretes a variety of biologically active factors, named “myokines”. Bostrom et al. have shown that PGC1-α overexpression in the muscles of mice stimulates an increase in expression of FNDC5, a membrane protein that is cleaved and secreted as a novel myokine, irisin [38]. Exercise increases circulating levels of irisin in mice and humans. Furthermore, increased irisin levels turn on thermogenesis by inducing the “browning” of white adipose tissue, thereby improving obesity and glucose homeostasis [38]. These results support the concept that skeletal muscle may also influence adipose tissue through circulating mediators such as myokines. Further studies are required to explore the role of myokines in the interaction between skeletal muscle and adipose tissue. Although, in this study, baseline skeletal muscle mass did not predict the changes in VFA during the follow-up period, longer follow-up period or larger number of participants may reveal baseline ALST mass as an independent risk factor for the development of visceral obesity.

There are several limitations to this study. First, neither muscle strength nor physical performance was considered when defining sarcopenia in the present study. However, because obesity itself is known to affect muscle strength and physical performance, a definition of sarcopenia based on those parameters may act as confounding factors. Second, although the DXA has been applied as one of the most commonly used clinical standards among several methods for studying body composition [39], it has some limitations. DXA cannot evaluate the muscle quality and composition in contrast with magnetic resonance image or CT [40]–[42]. In addition, because an important assumption made by DXA is that the hydration of fat-free mass is uniform, altered hydration status with aging may result in an error in the each amount of lean and fat compartment [43], [44]. Third, our cohort comprised well-functioning and generally healthy Asian men and women, which limits ability to generalize our study results to other populations with different ethnicity and characteristics. Finally, information on smoking status, alcohol consumption, and physical activity was self-reported, which may allow for a recall bias. On the other hand, strength of the current study is that it employs a sample of Korean men and women with a wide range of age. Furthermore, this longitudinal study was performed using well-defined inclusion and exclusion criteria, which may allow excluding the confounding effects of severe chronic diseases. In addition, we assessed skeletal muscle mass and visceral fat area using precise imaging techniques to detect sarcopenia and visceral obesity, such as DXA and CT, simultaneously at both baseline and follow-up examination.

In conclusion, this prospective cohort study demonstrated that increased visceral fat may result in future loss of skeletal muscle mass, although decreased skeletal muscle mass did not predict an increment of visceral fat in Korean men and women. These results may provide novel insight into sarcopenic obesity in an aging society.
